# fNIRS dataset during complex scene analysis

**DOI:** 10.3389/fnhum.2024.1329086

**Published:** 2024-03-21

**Authors:** Matthew Ning, Sudan Duwadi, Meryem A. Yücel, Alexander von Lühmann, David A. Boas, Kamal Sen

**Affiliations:** ^1^Department of Biomedical Engineering, Neurophotonics Center, Boston University, Boston, MA, United States; ^2^BIFOLD – Berlin Institute for the Foundations of Learning and Data, Berlin, Germany; ^3^Intelligent Biomedical Sensing (IBS) Lab, Technical University Berlin, Berlin, Germany

**Keywords:** open access, fNIRS, spatial attention, complex scene analysis, BCI

## Introduction

The human brain is an astonishingly powerful computational device, capable of feats yet to be matched by machines. One impressive example is the brain's ability to selectively attend to specific objects in a complex scene with multiple objects. For example, at a crowded cocktail party, we can look at a friend and hear what they are saying in the midst of other speakers, music, and background noise. Such multisensory filtering allows us to select and process important objects in a complex scene, a process known as Complex Scene Analysis (CSA) (Cherry, 2005; Haykin and Chen, 2005; McDermott, 2009). In stark contrast, millions of humans worldwide with disorders such as ADHD (Mihali et al., 2018; Fu et al., 2022), autism (Marco et al., 2011; Lolk, 2013), and hearing losses (Marrone et al., 2008) find such complex scenes confusing, overwhelming and debilitating. Thus, brain-computer interfaces (BCIs) and assistive devices for CSA have the potential to improve the quality of life for many humans.

Recently, we proposed a brain-inspired algorithm for auditory scene analysis based on a model of cortical neurons (Maddox et al., 2012; Dong et al., 2016; Chou et al., 2022). This algorithm has the potential to be applied in assistive devices for CSA. However, a critical piece of information required by the algorithm is the spatial location of the target stimulus. Thus, a portable and non-invasive technology that can decode the spatial location of the attended target stimulus during CSA would greatly facilitate the development of BCIs and assistive devices for CSA. In addition, a technology that provides insights into specific brain regions that play a significant role in decoding the attended location has the potential to advance our understanding of fundamental brain mechanisms underlying CSA in both normal and impaired humans.

Functional near-infrared spectroscopy (fNIRS) is a non-invasive neuroimaging technique that measures changes in oxygenated (HbO2) and deoxygenated hemoglobin (HbR) in the cerebral cortex (Chance et al., 1993; Girouard and Iadecola, 2006). Due to its portability and low cost, fNIRS has been used in Brain-Computer Interface (BCI) applications (Naseer and Hong, 2015). Previously, fNIRS has been applied to various aspects of auditory science such as classifying different sound categories (Hong and Santosa, 2016), identifying spatial locations of noise stimuli (Tian et al., 2021), characterizing hemodynamic responses to varying auditory stimuli (Pollonini et al., 2014; Steinmetzger et al., 2020; Luke et al., 2021), and investigating informational masking (Zhang et al., 2018, 2021). However, to date, fNIRS has not been applied to decode auditory and visual-spatial attention during CSA, and thus, no such dataset exists yet.

Here, we collected brain signals with fNIRS during the presentation of audio-visual stimuli in the presence of competing stimuli from multiple locations in order to mimic complex natural scenes. We targeted the dorsal frontoparietal network including frontal eye field (FEF) and intraparietal sulcus (IPS) as well as superior temporal gyrus/planum temporale (STG/PT), which were shown to be activated by auditory, visual, or audio-visual spatial tasks in fMRI (Shomstein and Yantis, 2006; Deouell et al., 2007; Wu et al., 2007; Smith et al., 2010; Van der Zwaag et al., 2011; Michalka et al., 2015, 2016) and simultaneous magnetoencephalography (MEG)/electroencephalogram (EEG) studies (Larson and Lee, 2013). We also recorded task performance by asking the subjects to identify the content of audio-visual stimuli that they were asked to attend to.

Both visual and auditory cues play an important role in CSA. One important observation made in the last few decades is that visual and auditory modalities can influence each other as shown in psychophysical, neurophysiological, and neuroimaging studies. These studies suggest the idea that perception combines features from both visual stimuli and auditory stimuli to form a single auditory-visual object (Bizley et al., 2016). Both auditory and visual features can contribute cross-modally to enhance auditory or visual object formation, which in turn, can enhance attention operating on auditory (Busse et al., 2005; Serences et al., 2005; Shomstein and Yantis, 2006; Maddox et al., 2015) or visual objects (Desimone and Duncan, 1995; Knudsen, 2007). Cross-modal influences have been demonstrated via activation or modulation in primary visual cortex by auditory stimuli (Petro et al., 2017), single-unit and local field potential modulation in primary auditory cortex by visual stimuli (Bizley et al., 2007; Kayser et al., 2008) and fMRI study on humans (Calvert et al., 1999, 2001; Laurienti et al., 2002; Martuzzi et al., 2006). Thus, employing auditory-visual objects to investigate CSA has the potential to reveal additional aspects of CSA beyond paradigms that utilize auditory stimuli alone, e.g., EEG-based auditory attention decoding algorithms. Our experimental paradigm uses multiple videos of individual speakers talking to simulate a cocktail party like setting. We also employ fNIRS to decode the attended location. fNIRS has higher spatial resolution and is less prone to movement artifacts compared to EEG. Moreover, fNIRs enables the measurement of brain activity under less restricted conditions compared to fMRI where subjects are required to be in a supine position. Thus, fNIRS has the potential to complement these other modalities to provide insight into CSA in more naturalistic settings.

This report provides an open-access fNIRS dataset that can be used to develop, test, and compare machine learning algorithms for classifying attended locations based on the fNIRS signals on a single trial basis. In total, we have collected ~990 trials in 11 subjects (30 trials in each condition for 3 conditions, 90 trials per subject for 11 subjects). Our sample preliminary analysis using Linear Discriminant Analysis (LDA) shows robust decoding of attended spatial location for more than half of our subjects for two class classification (individual subject accuracy provided in Table 1), demonstrating its potential for BCI systems in noisy environments. This dataset will be available for scientists and students for further analysis and exploration and enable posing and testing new hypotheses regarding spatial information processing. Analysis code for two-class and three-class classification performed in this paper is publicly available on GitHub at https://github.com/NSNC-Lab/fNIRS_DecodingSpatialAttention.

**Table 1:** Individual subject behavioral performance, classification accuracy, and statistics.

**Subjects**	**Audio and visual correctness (%)**	**Number of channels pruned (at SNR 1.5)**	**CV accuracy in percentage (2 class)**	**CV accuracy in percentage (3 class)**	**One way ANOVA statistics for three class features**			
					**ANOVA (F-statistic**, ***p*****-value)**	**Between class** ***p*****-values**		
						**Left (L) and Right (R)**	**Left (L) and Center (C)**	**Right (R) and Center (C)**
**8**	94.44	2	95	67	(35.86, 4.18e-16)	(L,R, 1.03e-4)	(L,C, 4.3e-17)	(R,C, 2.87e-5)
**10**	**48.89**	12	55	34	(4.58, 0.103)	(L,R, 0.2026)	(L,C, 0.4145)	(R, C, 0.0073)
**12**	93.33	18	92	84	(21.71,4.36e-10)	(L,R, 3.26e-10)	(L,C, 6.82e-5)	(R, C, 0.0612)
**13**	96.67	8	60	33	(1.19,0.305)	(L,R, 0.6038)	(L,C, 0.2766)	(R, C, 0.8217)
**14**	77.78	10	66	46	(18.61, 1.02e-8)	(L,R, 0.3267)	(L,C, 9.81e-9)	(R, C, 3.46e-4)
**15**	74.44	2	55	35	(30.39, 9.41e-14)	(L,R, 3.6e-7)	(L,C, 2.51e-14)	(R, C, 0.0174)
**16**	94.44	11	92	62	(24.3,3.38e-11)	(L,R, 1.9e-9)	(L,C, 0.9417)	(R, C, 1.06e-8)
**17**	80	**21**	68	33	(3.73,0.242)	(L,R, 0.0334)	(L,C, 0.9546)	(R, C, 0.0614)
**18**	96.67	**20**	65	33	(7.85,0.004)	(L,R, 2.305e-4)	(L,C, 0.1282)	(R, C, 0.1149)
**19**	96.67	7	83	42	(6.55,0.0015)	(L,R, 0.0058)	(L,C, 0.0043)	(R, C, 0.9998)
**20**	83.33	**25**	66	49	(0.07,0.93)	(L,R, 0.9943)	(L,C, 0.9521)	(R, C, 0.9363)
**21**	81.11	19	86	55	(1.09,0.336)	(L,R, 0.3164)	(L,C, 0.5264)	(R, C, 0.8999)
**22**	78.89	5	75	40	(11.98,6.63e-6)	(L,R, 6.27e-7)	(L,C, 0.0012)	(R, C, 0.3530)
**23**	78.89	18	65	46	(7.31,0.0007)	(L,R, 0.0016)	(L,C, 0.9591)	(R, C, 0.0033)
**24**	95.56	6	92	33	(9.06,0.0001)	(L,R, 2.51e-4)	(L,C, 0.9344)	(R, C, 0.0018)
**25**	**48.89**	12	50	30	(8.31,0.0003)	(L,R, 0.2033)	(L,C, 0.0299)	(R, C, 2.16e-4)

Subjects highlighted in green are included and the ones highlighted in red are excluded. The criteria for exclusion are the number of channels pruned at SNR 1.5 - if 20 or more channels are pruned, they are excluded from further analysis, and behavioral response correctness - if they scored below 70% in both face and transcript identification tasks. One way ANOVA test was performed to compare features for left, right, and center conditions. F-statistic and p-values are reported for ANOVA, and between class comparisons p-values are reported using Tukey-Kramer method in MATLAB.

## Methods

### Participants and demographics

Sixteen adults with normal hearing (age 19–48, 8 males and 8 females) were recruited for this study in accordance with the Institutional Review Board of Boston University, out of which only 11 (five males and six females) were included in analysis after exclusion criteria (exclusion criteria details are provided on Table 1 caption). A COVID-19 protocol was developed and strictly adhered to. Participants were screened to exclude those with history of major head trauma or neurological or psychiatric disorder, those who are taking psychoactive medication, those with facial or scalp eczema, those with abnormal or non-contact-corrected vision, and those with abnormal hearing or hearing impairment. Participants were briefed and consented before partaking in this study and were compensated for their time.

### Experimental paradigm

**Figure 1:**
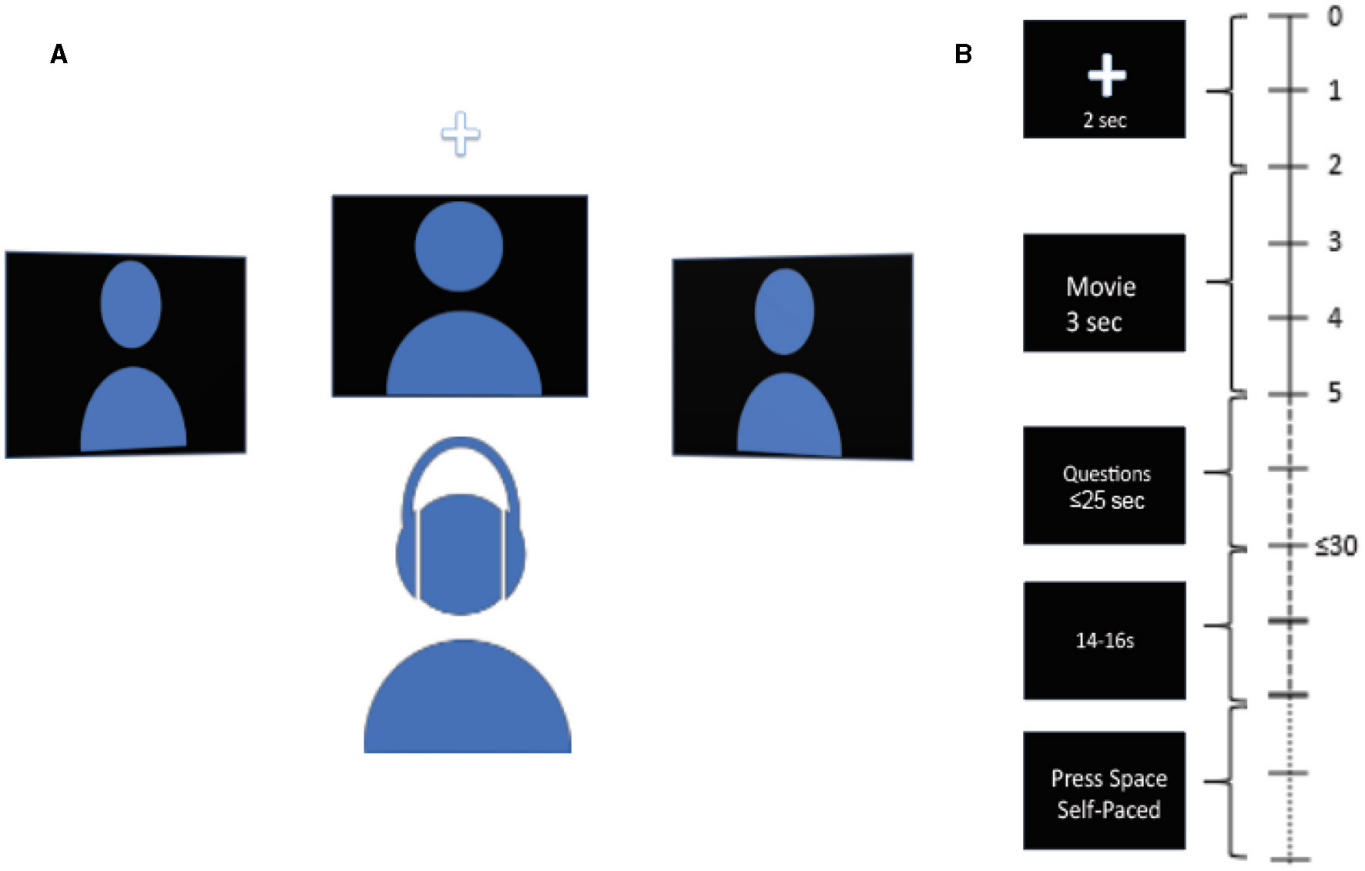
Schematic diagram of the experiment and trial. **(A)** Schematic diagram of experimental setup. Subject is seated in front of 3 spatially separated monitors, one at −45° to the left, one at 45° to the right, and one at 0° at the center. Audio is delivered via earphone. **(B)** Timeline of trial. The time at the right indicates the length of different parts of the trial. A cue in the form of a white cross accompanied by a pure ramping tone of 2 kHz randomly appears at one of the three locations to indicate the location of the target stimuli at the start of the trial. Next, 3 video clips are displayed simultaneously for 3 s. Next, two multiple-choice questions are displayed at the center monitor, to be answered with a keyboard. 1st question is to identify the face of the speaker, 2nd question is to identify the transcript spoken by the target speaker. Subject has up to 25 s to answer both questions. Upon the completion of the questions, a blank screen of jittered duration between 14 and 16 s follows. Next, instruction to press the space bar is displayed at the center monitor to begin the next trial.

Participants were seated in front of 3 monitors, located at locations equidistant from the subject: center or 0°, 45° to the left, and 45° to the right. The subjects were instructed to rest their chin on a chin rest throughout the experiment to discourage head movements. The subjects were asked to refrain from movements during the tasks except for answering the questions at the end of each trial. The auditory stimuli were delivered via an earphone (ER-1 Etymotic Research Inc.) with ear tips (E-A-RLink 3A Insert Eartips), and corresponding videos were displayed on the monitors. The videos used in the experiment are from AVSpeech, a publicly available dataset (Ephrat et al., 2018, https://looking-to-listen.github.io/avspeech/index.html). Videos were screened to include only those with English language and only those where speakers are alone in the videos. In the case there were two people in the video, strictly, only one person was talking. For each trial, a 2-s long audio-visual cue was delivered randomly at one of the 3 locations in the form of a white cross against a black background and a 2 kHz pure tone linearly ramped in the first 0.5 s. The subjects were instructed to listen to the cue and pay attention to the speaker at the cued location. The cue was followed by three videos, one for each location, one of which was the target speaker, and the remaining two were the maskers. The stimuli were followed by two multiple-choice questions on the center monitor, each question containing five possible choices. The first question was related to face identification, and the second was related to transcript identification. In the face identification task, the subjects were presented with five different faces and were tasked with correctly identifying the face of the target speaker shown in the video (“Who was the target speaker?”). In the transcript identification task, the subjects were presented with five different transcripts and were tasked with correctly identifying the transcript spoken by the target speaker in the video (“What was the target speaker saying?”). A trial was counted as correct if the participant correctly identified both the face and the transcript spoken by the target speaker. Upon the completion of two questions, a blank black screen of jittered duration with uniform distribution between 14 and 16 seconds appeared. Thereafter, an instruction to press the spacebar to begin the next trial was displayed on the center monitor. While the audio-visual cue and the video clip lasted 2 and 3 s respectively, the subjects had 20 s in total to answer both multiple-choice questions, but they could move on to the next trial by pressing the space bar immediately after they finished answering. Figure 1 shows the schematic of experiment setup and the timeline of trial progression.

### Data acquisition

fNIRS data were collected using continuous wave fNIRS (CW6, Techen System) using 690 nm and 830 nm wavelengths, with a 50 Hz sampling rate. Multiple channels were supported with frequency multiplexing. The fNIRS recording software was synced to the stimulus presentation with the Neurospec MMB70-817 triggerbox (Neurospec AG, Switzerland). Fifty-six cm Landmark Cap (EasyCap, Herrsching, Germany) was used for all the subjects.

### Measurements

#### fNIRS probe design

**Figure 2:**
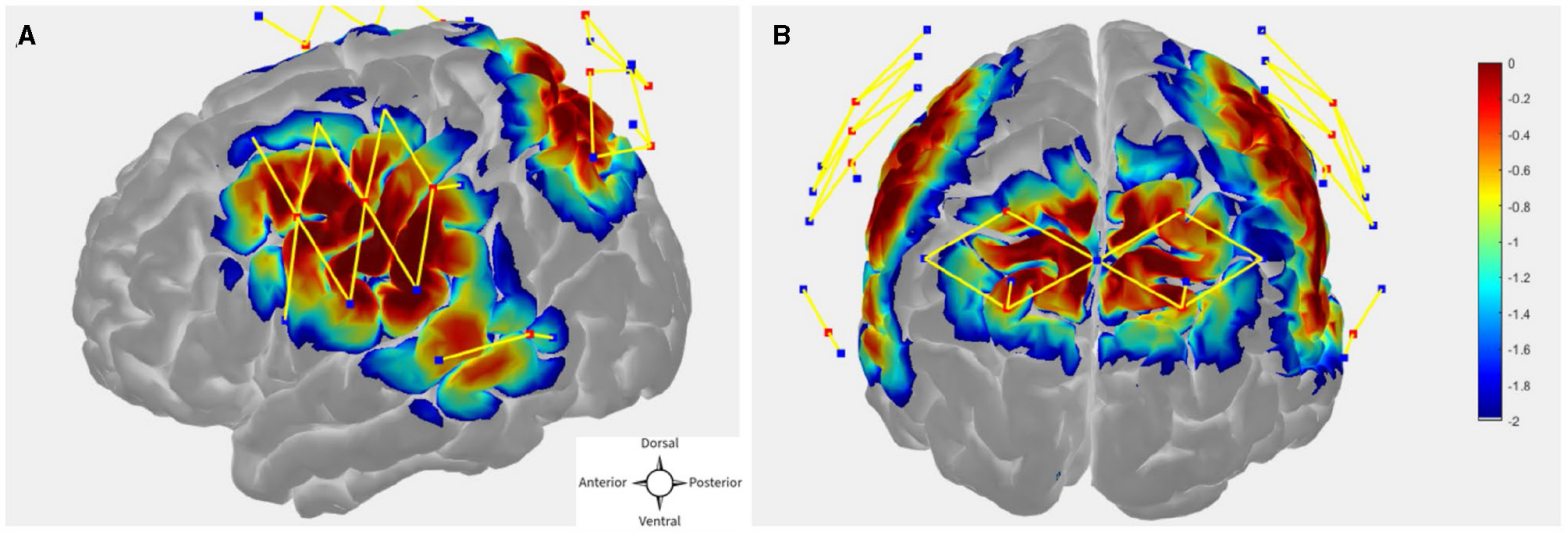
**(A, B)** fNIRS probe design with 12 sources, 17 regular separation detectors, 6 SS detectors. The sensitivity profile for the probe geometry is shown. Sources and detectors are denoted by red and blue square boxes respectively. The color scale represents the relative sensitivity in log10 units (Aasted et al., 2015). The longer yellow lines represent regular channels, and the shorter yellow lines represent short separation channels.

The probe (optode array) was designed in publicly available AtlasViewer software (https://github.com/BUNPC/AtlasViewer) (Aasted et al., 2015). The probe contains 12 sources, 17 long-separation detectors, and 6 short-separation detectors (SS), for a total of 30 long-separation channels (with an exception for one subject who had 14 sources, 19 long-separation detectors, and 8 short-separation detectors, for a total of 34 long-separation channels). The long-separation detectors were placed 30 mm from the sources whereas SS detectors were placed 8 mm from the sources. The probe covered the dorsal frontoparietal network including the frontal eye field (FEF) and intra-parietal sulcus (IPS) as well as the superior temporal gyrus/planum temporal (STG/PT). Figures 2A, B shows the sensitivity profile for the fNIRS probe geometry.

### Data structure and format

The fNIRS dataset consists of 90 trials each for 11 subjects. The behavioral dataset consists of recorded responses of the subject during each trial and actual answers to the questions asked to the subjects during each trial. fNIRS dataset is in “.snirf” format, and the behavioral dataset is in “.mat” format. Brain Imaging Data Structure (BIDS) compliant fNIRS dataset can be found on OpenNeuro (doi: 10.18112/openneuro.ds004830.v1.0.0). The behavioral dataset and the data structure details for both fNIRS and behavioral data can be found in the derivatives folder under “Experimental and Behavioral Dataset description.docx.”

## Preliminary analysis and data quality assessment

Preliminary fNIRS data preprocessing and analysis were done using publicly available Homer3 software (https://github.com/BUNPC/Homer3) (Huppert et al., 2009). Version of the Homer software used for preliminary analysis is included in GitHub along with custom scripts. Baseline classification analysis using LDA was done using custom code provided at: https://github.com/NSNC-Lab/fNIRS_DecodingSpatialAttention.

### Signal-to-noise ratio

We excluded subjects that had at least 20 channels pruned using a cutoff of SNR = 1.5, where SNR was estimated as the mean divided by the standard deviation of raw intensity of the fNIRS signal.

### fNIRS pre-processing

Raw light intensities were converted to optical densities using the mean signal as the arbitrary reference point. Motion artifacts in optical density (OD) were identified and corrected with targeted Principal Component Analysis (PCA) before applying criteria for automatic rejection of stimulus trials (Yücel et al., 2014). OD signals were band-pass filtered between 0.01 Hz and 0.5 Hz with a 3rd order zero-phase Butterworth filter. The filtered OD was converted to chromophore concentration changes. The differential path length factor was held fixed at 1, and the concentration unit is in Molar^*^mm (Scholkmann and Wolf, 2013). Finally, systemic physiological signal clutter was regressed out using a GLM with short-separation channels modeling the signal clutter (Gagnon et al., 2014; von Lühmann et al., 2020). Each regular channel was assigned an SS channel with the highest correlation (Gagnon et al., 2014).

For Hemodynamic response function (HRF) modeling, we fitted the GLM model to each subject. The GLM fitting here used two classes of regressors: HRF for each condition and the systemic signal clutter. The temporal basis functions used to model the HRF consisted of a sequence of 16 Gaussian functions, spaced 1 s apart, with a typical width of 1 s (Gagnon et al., 2011). This flexible model offers better fitting of the HRF shape at the expense of more parameter estimations than the typical canonical hemodynamic response function (Lindquist et al., 2009). Short-separation (SS) fNIRS signals were used as physiological regressors.

**Figure 3:**
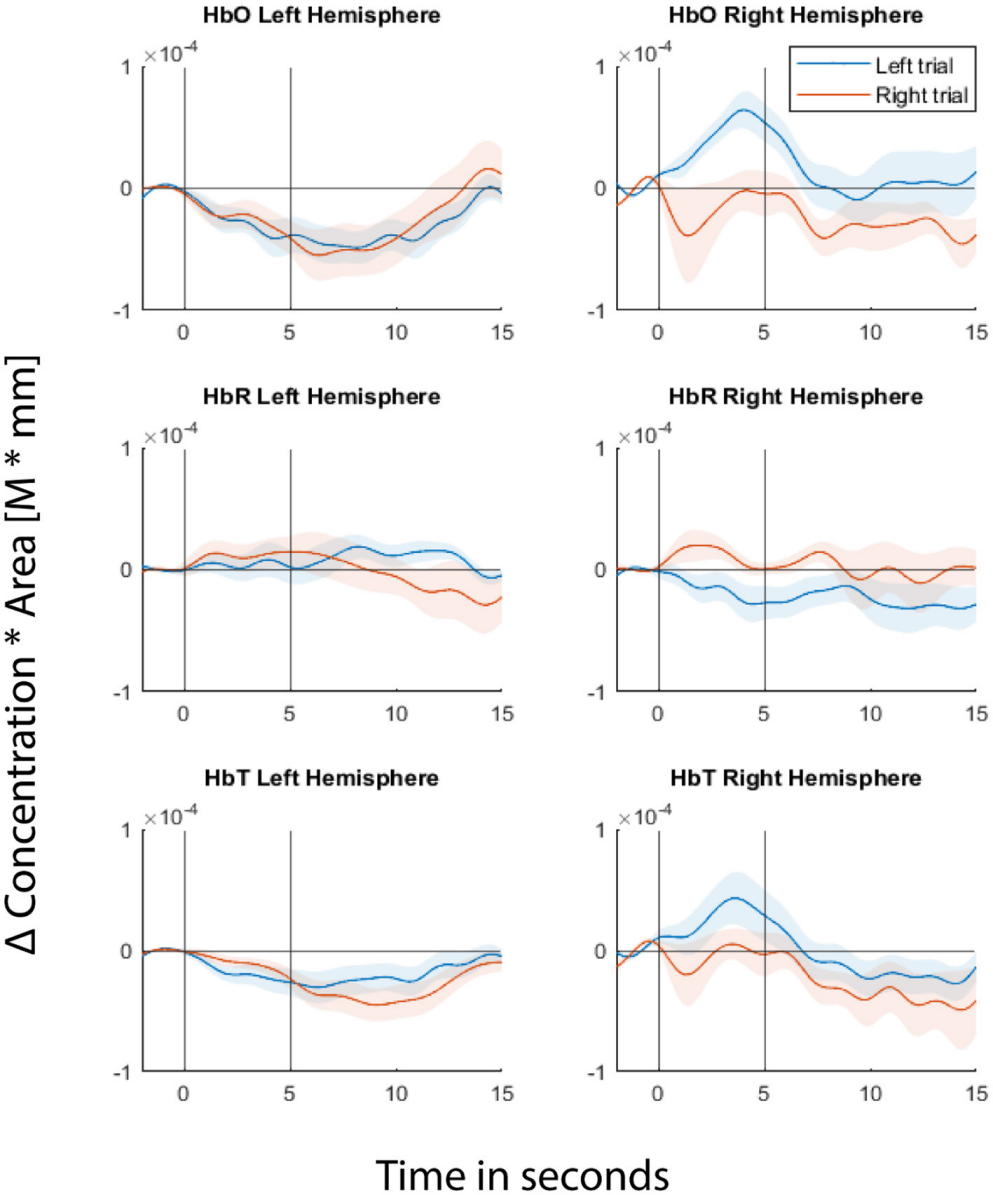
Hemodynamic Response Functions (HRFs) of two of the example channels, one from each hemisphere. Top panel represents Δ(HbO), middle panel represents Δ(HbR), and bottom panel represents Δ(HbT). Left column represents the channel from the left hemisphere and the right column represents the channel from the right hemisphere. Blue line represents GLM-fitted HRF responses to left stimuli and red line for right stimuli. Shaded regions represent 95% confidence intervals. The first black vertical line at 0th second represents the cue onset and the second vertical black line represents the movie offset. The movie starts at the 2nd second.

Fitting the GLM regression model to the entire data first before cross-validating the trials would result in information leakage (von Lühmann et al., 2020). In order to avoid leakage, we cross-validated both the GLM regression and classification steps. In each fold of the cross-validation, we fitted the GLM model to a training dataset and estimated regression coefficients using the Ordinary Least Squares (OLS) method. Then the short separation regression coefficients (SS coefficients) estimated from the training set are used for the test set where the individual trials are the difference between the measured fNIRS signals and the systemic physiological regressor weighted by the SS coefficients. Figure 3 shows the GLM fitted group level HRF for one of the example channels from each hemisphere for the readers to visualize the signals from which the features were extracted during classification.

Table 1 shows individual subject's data. It consists of behavioral data in terms of correctness of responses in both face and transcript identification tasks, number of channels pruned at SNR threshold of 1.5, individual subject's CV accuracy, and one way ANOVA results.

### Classification

**Figure 4:**
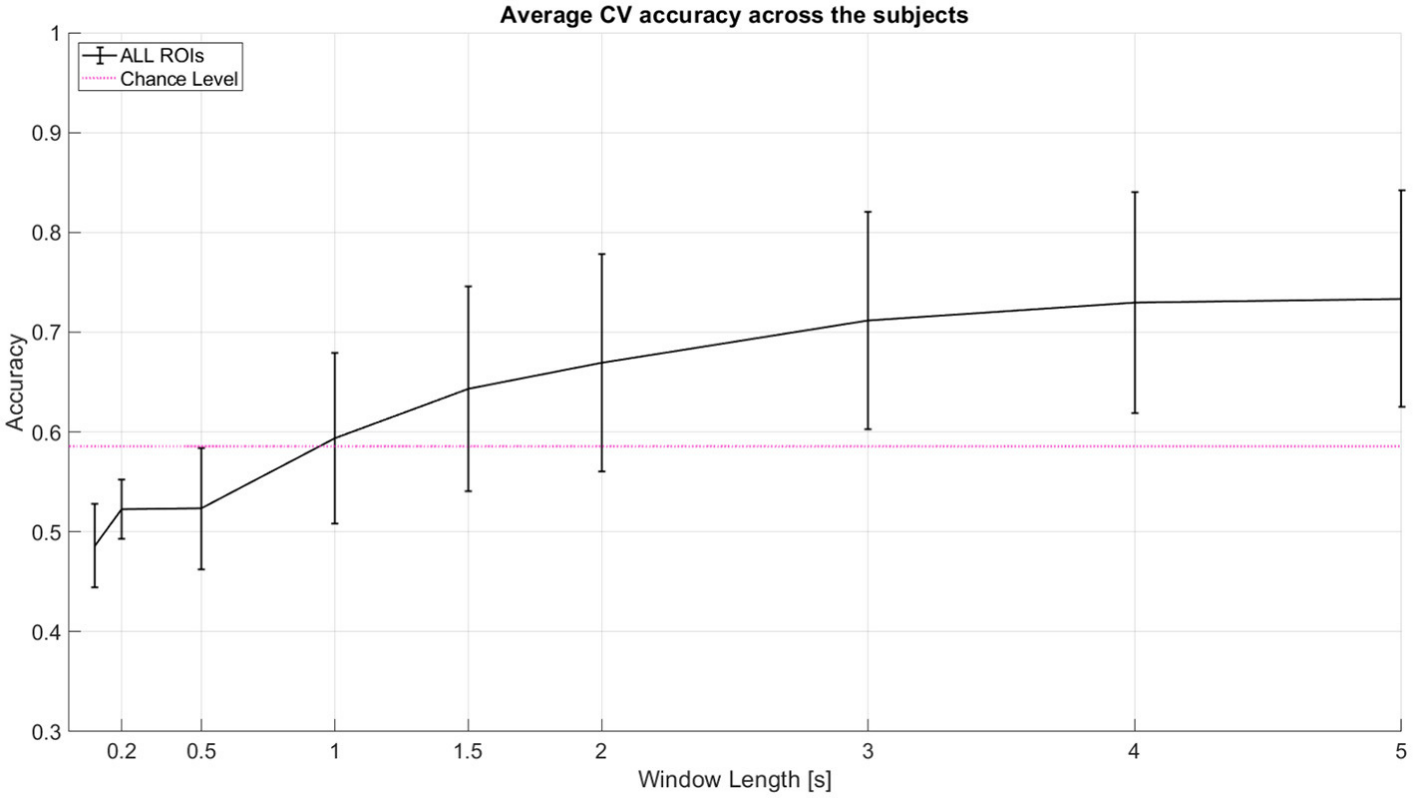
Average CV accuracy across 11 subjects when all regions of interest (ROI) are considered. All windows start at 0 s (cue onset). Decision window lengths tested here are 0 to 0.1, 0.2, 0.5, 1, 1.5, 2, 3, 4, and 5 s. The pink line indicates statistical significance for *p* = 0.05 using a two-tailed *t*-test and the given standard deviation from our cross-validation accuracies across subjects at window length 1 s. Error bars represent 95% confidence intervals (counting each subject's model performance accuracy as one sample point).

As a baseline analysis, we used LDA with linear shrinkage of the covariance matrix (Ledoit and Wolf, 2004). We tested 2-class classification between left (-45°) and right (45°) spatial locations. The features used were the area under the curve of the Hemodynamic Response Function (HRF) with total hemoglobin (HbT). The features were used from an incremental window where all windows start at 0th second (cue onset). Window lengths tested here are 0 to 0.1, 0.2, 0.5, 1, 1.5, 2, 3, 4, and 5 s. We performed 10 repetitions of 5-fold nested cross-validation (CV). Classifier test performance metric used is Cross Validation accuracy. We achieved a mean CV accuracy of ~73% for 2 classes and ~45% for 3 class classification at 5 seconds window. Table 1 shows the individual subject accuracy for both 2 class and 3 class classification. Figure 4 shows the average CV accuracy across the subjects when all of the channels were used (from all targeted regions of interest).

## Summary

Here, we presented an fNIRS dataset from 11 subjects, along with a behavioral dataset for decoding of attended audio-visual location. We include the custom MATLAB scripts for two-class and three-class classification. We also provide a link to the pre-processing code repository that includes any modification to the Master Homer3 package. This dataset can be used to train, test, and validate machine learning models for attention-based BCIs on a single trial basis.

## Data availability statement

The datasets presented in this study can be found in online repositories. The names of the repository/repositories and accession number(s) can be found in the article/supplementary material.

## Ethics statement

The studies involving humans were approved by the Boston Medical Center and Boston University Medical Campus Institutional Review Board. The studies were conducted in accordance with the local legislation and institutional requirements. The participants provided their written informed consent to participate in this study.

## Author contributions

MN: Data curation, Methodology, Software, Visualization, Writing – original draft, Writing – review & editing. SD: Software, Visualization, Writing – original draft, Writing – review & editing. MY: Methodology, Supervision, Writing – review & editing. AVL: Methodology, Writing – review & editing. DB: Methodology, Writing – review & editing. KS: Conceptualization, Supervision, Writing – review & editing.

## References

[B1] Aasted C. M. Yücel M. A. Cooper R. J. Dubb J. Tsuzuki D. Becerra L. . (2015). Anatomical guidance for functional near-infrared spectroscopy: AtlasViewer tutorial. Neurophotonics 2, 020801–020801. 10.1117/1.NPh.2.2.020801 26157991 PMC4478785

[B2] Bizley J. K. Maddox R. K. Lee A. K. C. (2016). Defining auditory-visual objects: behavioral tests and physiological mechanisms. Trends Neurosci. 39, 74–85. 10.1016/j.tins.2015.12.007 26775728 PMC4738154

[B3] Bizley J. K. Nodal F. R. Bajo V. M. Nelken I. King A. J. (2007). Physiological and anatomical evidence for multisensory interactions in auditory cortex. Cereb. Cortex 17, 2172–2189. 10.1093/cercor/bhl128 17135481 PMC7116518

[B4] Busse L. Roberts K. C. Crist R. E. Weissman D. H. Woldorff M. G. (2005). The spread of attention across modalities and space in a multisensory object. Proc. Nat. Acad. Sci. 102, 18751–18756. 10.1073/pnas.0507704102 16339900 PMC1317940

[B5] Calvert G. A. Brammer M. J. Bullmore E. T. Campbell R. Iversen S. D. David A. S. . (1999). Response amplification in sensory-specific cortices during crossmodal binding. NeuroReport 10, 2619–2623. 10.1097/00001756-199908200-00033 10574380

[B6] Calvert G. A. C. Iversen S. D. Brammer M. J. (2001). Detection of audio-visual integration sites in humans by application of electrophysiological criteria to the BOLD Effect. NeuroImage 14, 427–438. 10.1006/nimg.2001.0812 11467916

[B7] Chance B. Zhuang Z. UnAh C. Alter C. Lipton L. (1993). Cognition-activated low-frequency modulation of light absorption in human brain. Proc. Nat. Acad. Sci. U. S. A. 90, 3770–3774. 10.1073/pnas.90.8.3770 8475128 PMC46383

[B8] Cherry E. C. (2005). Some experiments on the recognition of speech, with one and with two ears. The J. Acous. Soc. Am. 25, 975–979. 10.1121/1.1907229

[B9] Chou K. F. Boyd A. D. Best V. Colburn H. S. Sen K. (2022). A biologically oriented algorithm for spatial sound segregation. Front. Neurosci. 16:1004071. 10.3389/fnins.2022.1004071 36312015 PMC9614053

[B10] Deouell L. Y. Heller A. S. Malach R. D'Esposito M. Knight R. T. (2007). Cerebral responses to change in spatial location of unattended sounds. Neuron 55, 985–996. 10.1016/j.neuron.2007.08.019 17880900

[B11] Desimone R. Duncan J. (1995). Neural mechanisms of selective visual attention. Ann. Rev. Neurosci. 18, 193–222. 10.1146/annurev.ne.18.030195.001205 7605061

[B12] Dong J. Colburn H. S. Sen K. (2016). Cortical transformation of spatial processing for solving the cocktail party problem: a computational model. eNeuro. 3:ENEURO.0086-15 2015. 10.1523/ENEURO.0086-15.2015 26866056 PMC4745179

[B13] Ephrat A. Mosseri I. Lang O. Dekel T. s Wilson K. Hassidim A. . (2018). Looking to listen at the cocktail party. ACM Trans. Graph. 37, 1–11. 10.1145/3197517.3201357

[B14] Fu T. Li B. Yin W. Huang S. Liu H. Song Y. . (2022). Sound localization and auditory selective attention in school-aged children with ADHD. Front. Neurosci. 16:1051585. 10.3389/fnins.2022.1051585 36620456 PMC9812578

[B15] Gagnon L. Perdue K. Greve D. N. Goldenholz D. Kaskhedikar G. Boas D. A. . (2011). Improved recovery of the hemodynamic response in diffuse optical imaging using short optode separations and state-space modeling. NeuroImage 56, 1362–1371. 10.1016/j.neuroimage.2011.03.001 21385616 PMC3085546

[B16] Gagnon L. Yücel M. A. Boas D. A. Cooper R. J. (2014). Further improvement in reducing superficial contamination in NIRS using double short separation measurements. NeuroImage 85, 127–135. 10.1016/j.neuroimage.2013.01.073 23403181 PMC3665655

[B17] Girouard H. Iadecola C. (2006). Neurovascular coupling in the normal brain and in hypertension, stroke, and Alzheimer disease. J. Appl. Physiol. 100, 328–335. 10.1152/japplphysiol.00966.2005 16357086

[B18] Haykin S. Chen Z. (2005). The cocktail party problem. Neural Comp. 17, 1875–1902. 10.1162/0899766054322964 15992485

[B19] Hong K. S. Santosa H. (2016). Decoding four different sound-categories in the auditory cortex using functional near-infrared spectroscopy. Hearing Res. 333, 157–166. 10.1016/j.heares.2016.01.009 26828741

[B20] Huppert T. J. Diamond S. G. Franceschini M. A. Boas D. A. (2009). HomER: a review of time-series analysis methods for near-infrared spectroscopy of the brain. Applied Optics 48, D280–D298. 10.1364/AO.48.00D280 19340120 PMC2761652

[B21] Kayser C. Petkov C. I. Logothetis N. K. (2008). Visual modulation of neurons in auditory cortex. Cereb Cortex 18, 1560–1574. 10.1093/cercor/bhm187 18180245

[B22] Knudsen E. I. (2007). Fundamental components of attention. Ann. Rev. Neurosci. 30, 57–78. 10.1146/annurev.neuro.30.051606.094256 17417935

[B23] Larson E. Lee A. K. C. (2013). The cortical dynamics underlying effective switching of auditory spatial attention. NeuroImage 64, 365–370. 10.1016/j.neuroimage.2012.09.006 22974974 PMC3508251

[B24] Laurienti J. Burdette J. H. Wallace M. T. Yen Y. F. Field A. S. Stein B. E. (2002). Deactivation of sensory-specific cortex by cross-modal stimuli. J. Cognit. Neurosci. 14, 420–429. 10.1162/089892902317361930 11970801

[B25] Ledoit O. Wolf M. (2004). A well-conditioned estimator for large-dimensional covariance matrices. J. Multiv. Anal. 88, 365–411. 10.1016/S0047-259X(03)00096-4

[B26] Lindquist M. A. Loh J. M. Atlas L. Y. Wager T. D. (2009). Modeling the hemodynamic response function in fMRI: efficiency, bias and mis-modeling. NeuroImage 45, S187–198. 10.1016/j.neuroimage.2008.10.065 19084070 PMC3318970

[B27] Lolk A. (2013). Diagnostic and Statistical Manual of Mental Disorders: DSM-5^*TM*^*, 5th Edn*. Arlington, VA: American Psychiatric Publishing, Inc., 947.

[B28] Luke R. Larson E. Shader M. J. Innes-Brown H. Van Yper L. Lee A. K. . (2021). Analysis methods for measuring passive auditory fNIRS responses generated by a block-design paradigm. Neurophotonics 8:025008. 10.1117/1.NPh.8.2.025008 34036117 PMC8140612

[B29] Maddox R. K. Atilgan H. Bizley J. K. Lee A. K. (2015). Auditory selective attention is enhanced by a task-irrelevant temporally coherent visual stimulus in human listeners. ELife 4:04995. 10.7554/eLife.04995 25654748 PMC4337603

[B30] Maddox R. K. Billimoria C. P. Perrone B. P. Shinn-Cunningham B. G. Sen K. (2012). Competing sound sources reveal spatial effects in cortical processing. PLoS Biol. 10:e1001319. 10.1371/journal.pbio.1001319 22563301 PMC3341327

[B31] Marco E. J. Hinkley L. B. Hill S. S. Nagarajan S. S. (2011). Sensory processing in autism: a review of neurophysiologic findings. Pediatric Res. 69, 48R–54R. 10.1203/PDR.0b013e3182130c54 21289533 PMC3086654

[B32] Marrone N. Mason C. R. Kidd G. (2008). Evaluating the benefit of hearing aids in solving the cocktail party problem. Trends Amp. 12, 300–315. 10.1177/1084713808325880 19010794 PMC2836772

[B33] Martuzzi R. Murray M. M. Michel C. M. Thiran J. P. P. Clarke S. Meuli R. A. (2006). Multisensory interactions within human primary cortices revealed by BOLD dynamics. Cereb. Cortex 17, 1672–1679. 10.1093/cercor/bhl077 16968869

[B34] McDermott J. H. (2009). The cocktail party problem. Curr. Biol. 19, R1024–R1027. 10.1016/j.cub.2009.09.005 19948136

[B35] Michalka S. W. Kong L. Rosen M. L. Shinn-Cunningham B. G. Somers D. C. (2015). Short-term memory for space and time flexibly recruit complementary sensory-biased frontal lobe attention networks. Neuron 87, 882–892. 10.1016/j.neuron.2015.07.028 26291168 PMC4545499

[B36] Michalka S. W. Rosen M. L. Kong L. Shinn-Cunningham B. G. Somers D. C. (2016). Auditory spatial coding flexibly recruits anterior, but not posterior, visuotopic parietal cortex. Cereb. Cortex 26, 1302–1308. 10.1093/cercor/bhv303 26656996 PMC4737613

[B37] Mihali A. Young A. G. Adler L. A. Halassa M. M. Ma W. J. (2018). A low-level perceptual correlate of behavioral and clinical deficits in ADHD. Comp. Psychiatry 2, 141–163. 10.1162/CPSY_a_00018 30381800 PMC6184361

[B38] Naseer N. Hong K. S. (2015). fNIRS-based brain-computer interfaces: a review. Front. Hum. Neurosci. 9:3. 10.3389/fnhum.2015.00003 25674060 PMC4309034

[B39] Petro L. S. Paton A. T. Muckli L. (2017). Contextual modulation of primary visual cortex by auditory signals. Philos. Trans. Royal Soc. B Biol. Sci. 372, 20160104. 10.1098/rstb.2016.0104 28044015 PMC5206272

[B40] Pollonini L. Olds C. Abaya H. Bortfeld H. Beauchamp M. S. Oghalai J. S. . (2014). Auditory cortex activation to natural speech and simulated cochlear implant speech measured with functional near-infrared spectroscopy. Hearing Res. 309, 84–93. 10.1016/j.heares.2013.11.007 24342740 PMC3939048

[B41] Scholkmann F. Wolf M. (2013). General equation for the differential pathlength factor of the frontal human head depending on wavelength and age. J. Biomed. Optics 18:105004. 10.1117/1.JBO.18.10.105004 24121731

[B42] Serences J. T. Shomstein S. Leber A. B. Golay X. Egeth H. E. Yantis S. . (2005). Coordination of voluntary and stimulus-driven attentional control in human cortex. Psychol. Sci.nce 16, 114–122. 10.1111/j.0956-7976.2005.00791.x 15686577

[B43] Shomstein S. Yantis S. (2006). Parietal cortex mediates voluntary control of spatial and nonspatial auditory attention. J. Neurosci. 26, 435–439. 10.1523/JNEUROSCI.4408-05.2006 16407540 PMC6674402

[B44] Smith D. V. Davis B. Niu K. Healy E. W. Bonilha L. Fridriksson J. . (2010). Spatial attention evokes similar activation patterns for visual and auditory stimuli. J. Cognit. Neurosci. 22, 347–361. 10.1162/jocn.2009.21241 19400684 PMC2846529

[B45] Steinmetzger K. Shen Z. Riedel H. Rupp A. (2020). Auditory cortex activity measured using functional near-infrared spectroscopy (fNIRS) appears to be susceptible to masking by cortical blood stealing. Hearing Res. 396, 108069. 10.1016/j.heares.2020.108069 32919177

[B46] Tian X. Liu Y. Guo Z. Cai J. Tang J. Chen F. . (2021). Cerebral representation of sound localization using functional near-infrared spectroscopy. Front. Neurosci. 15:739706. 10.3389/fnins.2021.739706 34970110 PMC8712652

[B47] Van der Zwaag W. Gentile G. Gruetter R. Spierer L. Clarke S. (2011). Where sound position influences sound object representations: a 7-T fMRI study. NeuroImage 54:32. 10.1016/j.neuroimage.2010.10.032 20965262

[B48] von Lühmann A. Ortega-Martinez A. Boas D. A. Yücel M. A. (2020). Using the general linear model to improve performance in fNIRS single trial analysis and classification: a perspective. Front. Hum. Neurosci. 14:30. 10.3389/fnhum.2020.00030 32132909 PMC7040364

[B49] Wu C. T. Weissman D. H. Roberts K. C. Woldorff M. G. (2007). The neural circuitry underlying the executive control of auditory spatial attention. Brain Res. 1134, 187–198. 10.1016/j.brainres.2006.11.088 17204249 PMC3130498

[B50] Yücel M. A. Selb J. Cooper R. J. Boas D. A. (2014). Targeted principle component analysis: a new motion artifact correction approach for near-infrared spectroscopy. J. Innov. Optical Health Sci. 7:1350066. 10.1142/S1793545813500661 25360181 PMC4211632

[B51] Zhang M. Alamatsaz N. Ihlefeld A. (2021). Hemodynamic responses link individual differences in informational masking to the vicinity of superior temporal gyrus. Front. Neurosci. 15:675326. 10.3389/fnins.2021.675326 34366772 PMC8339305

[B52] Zhang M. Mary Ying Y. L. Ihlefeld A. (2018). Spatial release from informational masking: evidence from functional near infrared spectroscopy. Trends Hearing 22:2331216518817464. 10.1177/2331216518817464 30558491 PMC6299332

